# Pangenome Evidence for Extensive Interdomain Horizontal Transfer Affecting Lineage Core and Shell Genes in Uncultured Planktonic Thaumarchaeota and Euryarchaeota

**DOI:** 10.1093/gbe/evu127

**Published:** 2014-06-12

**Authors:** Philippe Deschamps, Yvan Zivanovic, David Moreira, Francisco Rodriguez-Valera, Purificación López-García

**Affiliations:** ^1^Unité d’Ecologie, Systématique et Evolution, Centre National de la Recherche Scientifique (CNRS) and Université Paris-Sud, Orsay, France; ^2^Institut de Génétique et Microbiologie, Centre National de la Recherche Scientifique (CNRS) and Université Paris-Sud, Orsay, France; ^3^División de Microbiología, Universidad Miguel Hernández, San Juan de Alicante, Spain

**Keywords:** horizontal gene transfer, Thaumarchaeota, Euryarchaeota, ammonia-oxidizing archaea, uncultured archaea

## Abstract

Horizontal gene transfer (HGT) is an important force in evolution, which may lead, among other things, to the adaptation to new environments by the import of new metabolic functions. Recent studies based on phylogenetic analyses of a few genome fragments containing archaeal 16S rRNA genes and fosmid-end sequences from deep-sea metagenomic libraries have suggested that marine planktonic archaea could be affected by high HGT frequency. Likewise, a composite genome of an uncultured marine euryarchaeote showed high levels of gene sequence similarity to bacterial genes. In this work, we ask whether HGT is frequent and widespread in genomes of these marine archaea, and whether HGT is an ancient and/or recurrent phenomenon. To answer these questions, we sequenced 997 fosmid archaeal clones from metagenomic libraries of deep-Mediterranean waters (1,000 and 3,000 m depth) and built comprehensive pangenomes for planktonic Thaumarchaeota (Group I archaea) and Euryarchaeota belonging to the uncultured Groups II and III Euryarchaeota (GII/III-Euryarchaeota). Comparison with available reference genomes of Thaumarchaeota and a composite marine surface euryarchaeote genome allowed us to define sets of core, lineage-specific core, and shell gene ortholog clusters for the two archaeal lineages. Molecular phylogenetic analyses of all gene clusters showed that 23.9% of marine Thaumarchaeota genes and 29.7% of GII/III-Euryarchaeota genes had been horizontally acquired from bacteria. HGT is not only extensive and directional but also ongoing, with high HGT levels in lineage-specific core (ancient transfers) and shell (recent transfers) genes. Many of the acquired genes are related to metabolism and membrane biogenesis, suggesting an adaptive value for life in cold, oligotrophic oceans. We hypothesize that the acquisition of an important amount of foreign genes by the ancestors of these archaeal groups significantly contributed to their divergence and ecological success.

## Introduction

More than 1 decade ago, the exploration of microbial environmental diversity with molecular tools led to the discovery of several archaeal lineages in the oceanic water column. These were termed archaeal Groups I–IV according to the chronological order in which they were discovered (DeLong 1992; Fuhrman et al. 1992; Fuhrman and Davis 1997; López-García et al. 2001). Group I archaea branched at the base of the classical Crenarchaeota, one archaeal lineage so far composed exclusively of hyperthermophilic members, and raised increasing interest in subsequent years. It proved to be diverse and widespread not only in oceans, where it was particularly abundant at high depth (Karner et al. 2001; DeLong et al. 2006; Martin-Cuadrado et al. 2008), but also in freshwater and soils (Schleper et al. 2005; Leininger et al. 2006). The isolation of the first culturable member of this group from fish-tank sediments, the aerobic ammonia-oxidizing chemolithoautotroph *Nitrosopumilus maritimus* (Konneke et al. 2005), entailed the discovery that Group I archaea play a major ecological role as nitrifiers in the global nitrogen cycle (Nicol and Schleper 2006; Pester et al. 2011). Moreover, their distinct position in phylogenetic trees based on ribosomal proteins led to the proposal that the so-called Group I Crenarchaeota constituted an independent phylum, the Thaumarchaeota (Brochier-Armanet et al. 2008). Being widespread in oceans and soils, they were thought to be originally mesophilic. However, the discovery of early-branching thaumarchaeal lineages in hot springs and aquifers (de la Torre et al. 2008; Ragon et al. 2013) and their monophyly with the deep-branching hyperthermophilic Aigarchaeota and Korarchaeota (with which they form the well-supported TACK superphylum) suggest a thermophilic ancestry of the clade (Pester et al. 2011). Surprisingly, though several thaumarchaeal complete genome sequences are available, only that of *N. maritimus* (Walker et al. 2010) comes from free-living marine archaea and none from deep-sea plankton, where these archaea dominate but remain uncultured. Only recently some genomic sequences derived from single cells have been made available for the group (Rinke et al. 2013).

The environmental Groups II–IV belong to the Euryarchaeota and, compared with the Thaumarchaeota, remain much more enigmatic, lacking any cultured representative. Group IV Euryarchaeota appears to be rare; it branches at the base of the halophilic archaea and has been only detected in deep sea and cold, Arctic waters (López-García et al. 2001; Bano et al. 2004). The relatively more abundant marine Groups II and III Euryarchaeota (GII/III-Euryarchaeota) are sister clades that branch at the base of the cluster formed by *Aciduliprofundum boonei* and the Thermoplasmatales. Group II occurs throughout the water column, though peaks in the photic zone (Karner et al. 2001; DeLong et al. 2006; Ghai et al. 2010), whereas Group III is characteristic of deep waters (Fuhrman and Davis 1997; Martin-Cuadrado et al. 2008). Recently, a composite genome sequence grouping 4–6 strains of Group II archaea was assembled from surface seawater metagenomic sequences (Iverson et al. 2012). Its gene content suggested a motile, proteorhodopsin-based photo-heterotrophic lifestyle for these organisms. However, deep-sea Group II archaea diverge from surface dominant lineages and may lack proteorhodopsin (Frigaard et al. 2006). No genomic information exists for Group III archaea except for a few sequences from metagenomic fosmid libraries (DeLong et al. 2006; Martin-Cuadrado et al. 2008). Nonetheless, metagenomics and single-cell genomics are the most suitable ways to get functional and phylogenetic information from these uncultured groups.

Although most studies on marine archaea have focused on their potential metabolism and ecology, earlier preliminary work suggested that horizontal gene transfer (HGT) from distant donors might have been important in the evolution of these archaeal groups. Thus, initial phylogenetic analyses of 22 fosmid clones (30- to 40-kbp long) containing 16S rRNA genes of uncultured deep-sea Thaumarchaeota (Group I) and GII-Euryarchaeota revealed a notable proportion of genes of bacterial origin (López-García et al. 2004; Brochier-Armanet et al. 2011). Further phylogenetic analysis of fosmid-end sequences from several thousand clones in deep-sea metagenomic libraries suggested that HGT from bacteria could be important in the rest of the genome (Brochier-Armanet et al. 2011), but the archaeal nature of those fosmid clones and the directionality of gene transfer remained to be unambiguously determined. On similar lines, a basic local alignment search tool (BLAST)-based comparison of the surface composite Group II genome showed that a significant proportion of genes had similarity with bacterial genes (Iverson et al. 2012). However, BLAST analyses are far from conclusive (Koski and Golding 2001). Therefore, although these studies suggested extensive directional bacteria-to-archaea gene transfer, this remained to be explicitly shown at a whole-genome level. The occurrence of potential high interdomain HGT levels opened also questions as to when those transfers took place and what their selective advantage might be. If they were ancient and predated the ancestor of the two archaeal lineages, did they play a role in their early diversification by, for instance, allowing the colonization of new environments? If, on the contrary, those HGT events are recent and not shared by different archaeal strains, do archaea have particular ability to gain and loss foreign genes and why?

To try to answer to those questions, we first seek to confirm whether members of these uncultured marine archaeal lineages have acquired significant proportions of “long-distance”-transferred genes at genome-wide level and, second, we ask whether putative transferred genes affected differentially core and shell genes (ancient vs. recent acquisitions) or whether HGT was an ongoing process. To answer, we sequenced 997 fosmid archaeal clones from deep-Mediterranean metagenomic libraries and built comprehensive composite gene complements for both, Thaumarchaeota and GII/III-Euryarchaeota, defining sets of core, lineage-specific core, and shell genes within the two archaeal pangenomes. We show by systematic and curated molecular phylogenetic analyses that a substantial fraction of genes in the lineage-specific core and shell gene sets was acquired from bacteria, implying directional and ongoing bacteria-to-archaea HGT.

## Materials and Methods

### Selection and Sequencing of Fosmid Clones from Deep-Mediterranean Metagenomic Libraries

The archaeal fosmids were retrieved from two deep-sea Mediterranean fosmid libraries constructed using DNA purified from the 0.2–5 µm cell diameter plankton fraction of, respectively, 3,000 m-deep Ionian Sea (36°20′N; 15°39′E) and 1,000 m-deep Adriatic Sea (41°36′N; 17°22′E) waters (Martin-Cuadrado et al. 2007, 2008). The two extremities of inserts were sequenced for 12,774 fosmids per library, and BLAST and phylogenetic analyses were subsequently carried out for each fosmid-end sequence and used to identify genes of putative archaeal nature, as previously described (Brochier-Armanet et al. 2011). These were genes of widespread distribution in archaea and either absent in bacteria or present but forming a monophyletic clade to the exclusion of all archaea. On the basis of the archaeal nature of fosmid-end sequences, we selected and sequenced a total of 997 archaeal fosmids, 545 out of which were ascribed to the Thaumarchaeota (formerly Group I Crenarchaeota) and 452 to the Euryarchaeota (Groups II/III, summarized in the following as GII-Euryarchaeota) (table 1). Selected fosmid clones were grown in lysogeny broth medium + chloramphenicol and multicopy fosmid production induced as described by the manufacturer of the CopyControl Fosmid Library Production Kit (Epicentre). Cultures of 96 fosmid clones were pooled together and DNA extracted using the QIAprep Spin Miniprep Kit (Qiagen, Valencia, CA). Fosmids were 454 pyrosequenced using Titanium chemistry in pools of 200 fosmids per run (Beckman Coulter Genomics, Denver, CO), leading to an average coverage per fosmid of 54×.

**Table 1:** Deep-Mediterranean Archaeal Fosmid Sequence Data and Distribution of OG Clusters According to Their Class of Origin Based on Manually Inspected Phylogenetic Trees

	Thaumarchaeota	Euryarchaeota GII/III
Number of fosmids	545	452
Total sequence (bp)	19,717,229	16,310,525
Mean fosmid insert length (bp)	36,178	36,085
GC content (%)	47.13	54.82
rRNAs (5S, 16S, 23S)	42	28
tRNAs	610	489
Mean of 40 single-copy genes^a^	16.5	9.3
ORFs ≥ 90 nt	150,170	164,605
Number of annotated genes	23,665	13,227
Classes of gene clusters		
Core (archaeal + universal)	629	552
Specific core—non-HGT	416	288
Specific core—early HGT	290	416
Early HGT shared with *Nitrososphaera*	196	—
Shell—non-HGT^b^	452	1,256
Shell—late HGT	311	1,015
Total OG clusters	2,098	3,527
Orphan genes	416	1,293

^a^Details on single-copy gene numbers are shown in supplementary figure S1, Supplementary Material online.

^b^Predicted genes with homologs only in other deep-Mediterranean fosmids or showing among 1–3 similar hits in the database.

### Sequence Statistics, Annotation, and Functional Classification of Genes

Tetra- and pentanucleotide frequencies were computed for each fosmid nucleotide sequence with the TETRA package (Teeling, Waldmann, et al. 2004). Subsequently, *z* scores data values derived from the frequency matrix were used to conduct principal component analysis (PCA) (Raychaudhuri et al. 2000) using the MeV program (Saeed et al. 2003). Each contig was individually processed for gene identification and annotation as follows. We identified all open-reading frames (ORFs) ≥ 30 amino acids (aa) using the bacterial, archaeal, and plant plastid code (transl_table=11, see http://www.ncbi.nlm.nih.gov/Taxonomy/taxonomyhome.html/index.cgi?chapter=cgencodes, last accessed June 25, 2014). In parallel, candidate coding DNA sequences (CDSs) were defined using Prokaryotic Dynamic Programming Genefinding Algorithm (version 2.60, see http://prodigal.ornl.gov/, last accessed June 25, 2014) (Hyatt et al. 2010). The two sets were thereafter matched, followed by automated annotation and CDS prediction corrections as follows. First, all ORFs were submitted to similarity search using BLASTP (Altschul et al. 1997) against the RefSeq_protein nonredundant database (GenBank, spanning 11/11-04/13 versions), SWISSPROT release 57.11, the clusters of orthologous group (COG) databases (COG + KOG, seven eukaryotic genomes), and KEGG pathways database (Kanehisa Laboratories, Release 2012-11-12, see ftp://ftp.bioinformatics.jp/). Motif searches were performed in the conserved domain databases (CDD): CDD.v.2.17, Pfam.v.23.0, SMART.v5.1, COG.v.1.0, KOG.v.1.0, TIGR.v.8.0, and PRK.v.4.0 using RPS-BLAST (Marchler-Bauer et al. 2005). Predicted CDS having matches in the RefSeq database with an *e* value ≤ 1 e−10 were validated as genes. Among these, CDS matching orphan RefSeq genes (i.e., hypothetical proteins) were examined to determine whether they matched a COG functional category or contained any known motif in CDD databases. In such cases, the accepted annotation was switched to that of the relevant match, provided their BLASTP and RPS-BLAST *e* values remained below the 1 e−05 threshold. Small (<100 aa) orphan genes and predicted genes overlapping structural RNA genes were stripped off our gene list. Small ORFs ruled out in the CDS prediction step were checked for significant matches in RefSeq, COG, and CDD databases (with similar *e* value thresholds as above). These small ORF candidates were validated as genes provided that they did not overlap any other gene having high similarity in searched databases. Finally, tRNAs were identified using tRNA-scanSE (Lowe and Eddy 1997), and ribosomal RNA genes were identified with rRNA_hmm_fs/hmmsearch 3.0 (Huang et al. 2009). Annotated fosmids have been deposited in GenBank with accession numbers KF900301–KF901297.

### Taxonomic Affiliation of Archaeal Fosmids

Genes from annotated fosmids were initially tagged according to the taxonomy of their best BLASTP hit in the RefSeq database. To class fosmids according to their most probable phylogenetic origin, the percent of genes affiliating to different broad taxonomic categories (Archaea, Bacteria, Eucarya, viruses, Crenarchaeota, Euryarchaeota, Thaumarchaeota, Nanoarchaeota, Korarchaeota, and unclassified archaea) was calculated for each fosmid and the resulting data matrix was processed with a quality cluster method (QT_Clust) (Heyer et al. 1999) with MeV (Multiexperiment Viewer; http://www.tm4.org/mev.html, last accessed June 25, 2014) (Saeed et al. 2003). This provided a preliminary ascription of fosmids to different taxonomic groups. Subsequently, the affiliation of fosmids initially classed as archaeal was refined by phylogenetic analysis of all individual genes (see below).

### Definition of Core, Lineage-Specific Core, and Shell Genes in Archaeal Pangenomes

**Fig. 1.—:**
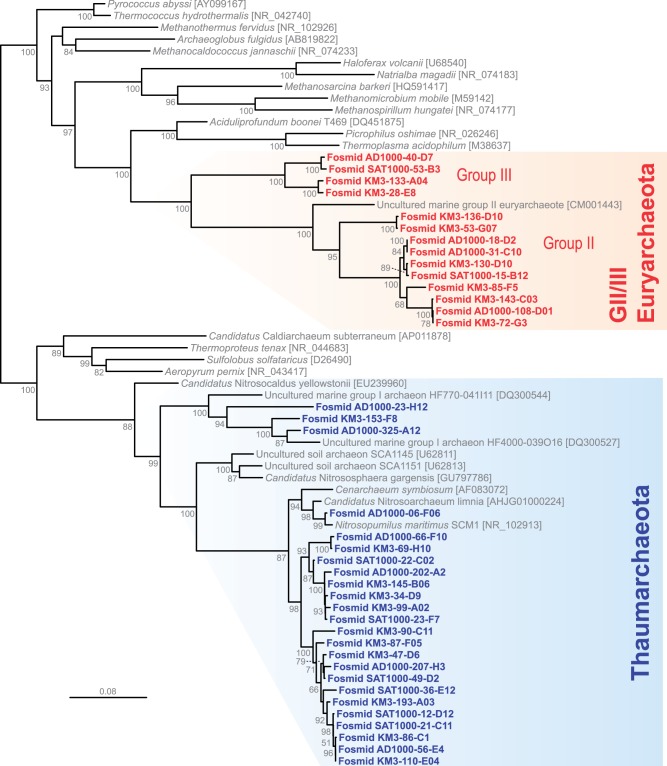
Phylogenetic tree of 16S rRNA genes present in deep-Mediterranean archaeal fosmids from Ionian (KM3; 3,000 m depth) and Adriatic (AD1000; 1,000 m depth) metagenomic libraries. Colored areas correspond to the lineages for which pangenome gene complements have been defined. Note: Several fosmids were identified in a previous study (Brochier-Armanet et al. 2011). The tree was reconstructed using 1,343 conserved nucleotide positions.

Given the relative high coverage obtained for closely related deep-sea Thaumarchaeota and GII/III-Euryarchaeota lineages with, respectively, approximately 15 and 9 complete genomes as estimated from single-copy genes (see table 1, fig. 1, and supplementary fig. S1, Supplementary Material online), we defined orthologous gene (OG) clusters for our deep-sea Thaumarchaeota and GII/III-Euryarchaeota fosmids which, collectively, were considered to represent their respective pangenomes. Subsequently, we classified them into core archaeal genes (universal or universal in archaea), lineage-specific core genes (genes shared by, respectively, the Thaumarchaeota and GII/III-Euryarchaeota), and shell or accessory genes in each lineage. To do so, we compiled genes sets from our marine Thaumarchaeota and GII/III-Euryarchaeota metagenomic fosmids together with those of the respective closest phylogenetic relatives for which genome sequences were available: *N. maritimus* SCM1 (NC_010085), *Cenarchaeum symbiosum* A (NC_014820), and *Nitrosoarchaeum limnia* SFB1 (Blainey et al. 2011) for Thaumarchaeota, and the composite genome built from surface seawater metagenome (CM001443.1) for GII-Euryarchaeota (Iverson et al. 2012). Core archaeal genes were defined whenever orthologous clusters from all representative Thaumarchaeota and Euryarchaeota genomes were present. Thaumarchaeota or GII/III-Euryarchaeota-specific core genes were defined when clusters from all Thaumarchaeota reference genomes (or all but one) or the surface marine GII-euryarchaeote were present in our fosmids according to the same similarity and alignment length criteria as above. Accessory or shell genes corresponded to gene clusters that were not present in all archaea, all the Thaumarchaeota, or all the Euryarchaeota reference genomes, genes having only 1–3 hits in the database or genes that lacked homologs in archaea but not in other life domains. This initial classification in broad categories was manually refined based on phylogenetic analyses.

### Phylogenetic Analyses, Refinement of Orthologous Clusters, and Identification of HGT Events

Truncated genes at fosmid ends or sequences containing ambiguous positions (Ns) may escape the above criteria for automatic clustering and lead to a number of misclassifications and to the definition of a few erroneous clusters that need rectification. In addition, similarity-based analyses of cluster taxonomic affiliation are only indicative and need to be validated by proper phylogenetic analyses. For this purpose, all the predicted ORF protein sequences in fosmids were included in a local genome database together with proteomes of 120 archaea (encompassing genomes, metagenomes, and environmental fosmids), 297 bacteria spanning the diversity of bacterial phyla, and 120 eukaryotes. We used an automated pipeline to reconstruct phylogenetic trees for each ortholog cluster and each nonclustered gene. Genes were compared (BLASTP) with our local database and aligned with their best BLASTP hits (maximum 250 hits per gene; *e* value exclusion threshold, 1 e−5) using MAFFT with default parameters (Katoh et al. 2005). Each alignment was subsequently inspected, and misaligned sites or sites with more than 20% gaps were removed using BMGE (Criscuolo and Gribaldo 2010). Phylogenetic trees were computed from the resulting alignments using FastTree with default parameters and an automatic choice of substitution model (Price et al. 2009). Only data sets containing a minimum of four homologous sequences were retained for phylogenetic reconstruction. Trees were inspected manually to correct or refine the core/shell classification and to determine the origin of the transfers. The phylogenetic origin of a cluster or a single gene was determined from the closest neighbors in the tree at two levels of precision depending on the quality of the phylogenetic signal retained: Domains (Eucarya, Archaea, and Bacteria) or phyla/classes (Euryarchaeota, Crenarchaeota, and Thaumarchaeota for the Archaea; Alpha-, Beta-, Gamma-, Delta-Proteobacteria, Firmicutes, Chlamydia, CFB, Actinobacteria, Acidobacteria, and Cyanobacteria for the Bacteria). We inferred bacteria-to-archaea HGT events for a given gene 1) when phylogenetic trees reproduced with reasonable support the overall monophyly of recognized bacterial phyla (though local zones of low resolution might sometimes occur, as well as limited HGT among bacteria) and Thaumarchaeota or GII/III-Euryarchaeota genes formed a monophyletic group branching within a bacterial phylum or group of monophyletic phyla and/or 2) when Thaumarchaeota or GII/III-Euryarchaeota appeared alone to the exclusion of other archaea forming a monophyletic group among many bacterial phyla including many sequences. Poorly resolved trees (when the phylogenetic signal of a given gene was too low and bacterial and archaeal sequences were intermixed), trees showing a very high level of HGT among bacteria or trees with few archaeal and bacterial members were (conservatively) excluded from our analysis. The manual analysis of our trees led to a more reliable list of clusters, which were classified according to their phylogenetic origin and distribution (table 1 and fig. 3). A proportion of genes had no similarity in the database (orphans) (table 1). To verify the prediction that HT-genes present in marine Thaumarchaeota were also shared by soil Thaumarchaeota, we included the genome of *Nitrososphaera gargensis* (Spang et al. 2012) in our database, looked for homologs to the HT-genes that we had identified in our thaumarchaeal pangenome and reconstructed phylogenetic trees of those genes as above.

### Synteny Analysis and Horizontally Transferred Genes

Synteny blocks in archaeal fosmids were defined as arrays of one of more contiguous genes of same origin class (archaeal core, lineage-specific core, early HT-genes, late HT-genes, and others, including the remaining predicted genes without homologs in the database). Each gene or synteny block is flanked by blocks of one or two different origins. Because we consider five possible origin gene classes for Thaumarchaeota or GII/III-Euryarchaeota, there are 15 (5 + 4 + 3 + 2 + 1) different possibilities for any synteny block (or gene) to be bounded. Bounding-couple occurrence was compiled for each synteny block in Thaumarchaeota and GII/III-Euryarchaeota fosmids separately and the corresponding data matrix subjected to hierarchical clustering analysis (Eisen 1998) using the MeV package (Saeed et al. 2003).

### Codon Usage and Codon Adaptation Index Analysis of HT-Genes

The codon adaptation index (CAI) for a total of 26,678 genes acquired through HGT by Thaumarchaeota and GII/III-Euryarchaeota was calculated as follows: 1,244 ribosomal protein genes (459 from GII-Euryarchaeota and 785 from Thaumarchaeota fosmids) were first selected as a reference pool of highly expressed genes, either together or in groups of similar origin, and their codon usage table calculated using the cusp program from the EMBOSS suite, version 6.5.7 (Rice et al. 2000). The CAI for all genes was then calculated with the three sets of ribosomal genes codon usage tables (Thaumarchaeota, Euryarchaeota, or Thaum + Euryarchaeota) serving as reference with the CAI program (EMBOSS). Codon usage values were then submitted to PCA analysis (Raychaudhuri et al. 2000) using MeV (Saeed et al. 2003).

## Results

### Metagenomic Fosmid Sequences and Functional Classification of Genes in Archaeal Pangenomes

**Fig. 2.—:**
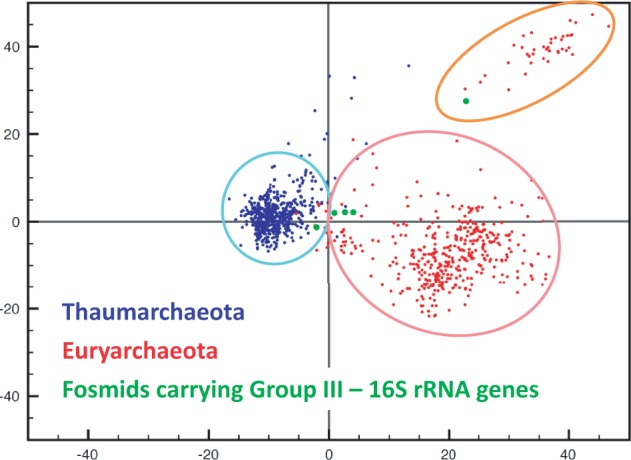
PCA of tetranucleotide frequencies in sequenced fosmids for Thaumarchaeota (blue) and GII/III-Euryarchaeota (red).

We obtained complete sequences of 545 and 452 fosmid clones from metagenomic libraries of deep-Mediterranean plankton (Ionian and Adriatic Seas at, respectively, 3,000 and 1,000 m depth) clearly affiliated with, respectively, Thaumarchaeota and GII/III-Euryarchaeota. Phylogenetic ascription was initially based on the phylogeny of genes located at both insert ends (Brochier-Armanet et al. 2011) and, subsequently, confirmed or corrected based on the phylogeny of all the genes that they contained (see below). Only high-quality, full-fosmid sequences showing no indication of potential chimerism (e.g., frameshifts, truncated genes, or unmixed distribution of archaeal and bacterial genes in two fosmid regions) were retained for this study. Details about the genomic sequences generated are given in table 1. Because Thaumarchaeota and GII/III-Euryarchaeota seem to have a single copy of rRNA genes (this is the case in all sequenced genomes of Thaumarchaeota as well as the Thermoplasmatales and *A. boonei*, the closest relatives of GII/III-Euryarchaeota) (Moreira et al. 2004), the number of archaeal genomes sequenced could be estimated at, respectively, 14 and 9, based on the number of rRNA gene copies identified. These values were in good agreement with estimates obtained from a collection of 40 additional genes typically found in single copy in prokaryotic genomes (Creevey et al. 2011), 16.5 and 9.3, respectively (supplementary fig. S1, Supplementary Material online, and table 1). The identification of similar gene counts for all those single-copy genes additionally suggests that those archaeal genome equivalents had complete (or nearly so) coverage in our libraries in terms of gene content. However, the assembly of individual genomes was not possible due to the within-group archaeal diversity captured by the fosmids (see below; fig. 1). To try to bin fosmids within different phylogenetic groups, we analyzed tetranucleotide and pentanucleotide frequencies, which are often used for the assignment of genome fragments to distinct groups (Teeling, Meyerdierks, et al. 2004). A PCA of tetranucleotide frequencies showed a clear separation of Thaumarchaeota and Euryarchaeota fosmids (fig. 2). Thaumarchaeota fosmids formed a tight cluster, and different subgroups were not distinguishable. Euryarchaeota fosmids formed a much more dispersed cloud, with a small cluster of fosmids loosely segregating from the main cloud. However, contrary to our initial expectations, this smaller cluster does not correspond to GIII-Euryarchaeota, because fosmids containing 16S rRNA genes of GIII-Euryarchaeota fell in the two clouds (mostly in the bigger cloud). The eccentricity of those clones is so far unclear; they might contain genomic islands with biased GC content/codon usage or differentially expressed genes. At any rate, the lack of reference genomes for Euryarchaeota, especially for GIII, prevents to attribute confidently fosmids without 16S rRNA genes to any of the two groups. Consequently, for the purpose of this work, and because GII and GIII are clearly monophyletic, we considered a collective GII + GIII-Euryarchaeota pangenome for the rest of our phylogenetic study.

Phylogenetic analyses of single-copy conserved marker genes, such as 16S rRNA genes, EF-2, or ribosomal protein S2 (fig. 1 and supplementary fig. S2, Supplementary Material online), revealed a diversity of deep-sea Mediterranean Thaumarchaeota and GII/III-Euryarchaeota congruent with previous studies from the same samples (Martin-Cuadrado et al. 2008). Thaumarchaeal fosmids were vastly dominated by a few closely related operational taxonomic units (OTUs) forming a sister, though distant, clade to the *Cenarchaeum–Nitrosopumilus* cluster (Martin-Cuadrado et al. 2008) (fig. 1). This clade is widely represented in the deep ocean and therefore represents a clade of truly deep planktonic Thaumarchaeota, in contrast to the *Cenarchaeum*–*Nitrosopumilus* clade, which might correspond to organisms best thriving in other marine niches (e.g., sponges, sediment, and surface waters). In addition, a minor proportion of fosmids corresponded to a basal, typically marine lineage branching out earlier than the soil Thaumarchaeota cluster, sometimes referred to as the ALOHA or 1A group (DeLong et al. 2006; Martin-Cuadrado et al. 2008; Pester et al. 2011) (fig. 1 and supplementary fig. S2, Supplementary Material online). Euryarchaeal fosmids encompassed a series of OTUs distantly related to the surface GII-euryarchaeote composite genome and to a clade of more basal sequences defining the deep-sea GIII-Euryarchaeota (fig. 1 and supplementary fig. S2, Supplementary Material online). Although both marine Thaumarchaeota and GII/III-Euryarchaeota represent relatively diverse groups, for the purpose of this study we considered deep-Mediterranean fosmid-derived pangenomes as representative of the two archaeal lineages. In the case of the Thaumarchaeota, we decided to include the deep-branching marine lineage to test whether some gene transfers were shared by all the marine Thaumarchaeota identified so far.

Genes for deep-Mediterranean Thaumarchaeota and GII/III-Euryarchaeota lineages were annotated and classified according to their predicted function in COG categories and KEGG classes (supplementary figs. S3 and S4, Supplementary Material online). In the case of Thaumarchaeota, genes encoding ammonium monooxygenase subunits and ammonium transporters were found to be present in equivalent numbers to single-copy genes in our Mediterranean fosmids (supplementary fig. S5, Supplementary Material online). Likewise, urease and urea transport genes were found in similar proportions. This strongly suggests that deep-Mediterranean Thaumarchaeota are ammonia oxidizers and that, similarly to their deep Arctic relatives, they utilize urea to fuel nitrification (Alonso-Saez et al. 2012). Urea degradation seems to be a metabolic feature of deep-sea Thaumarchaeota thriving in highly oligotrophic conditions, irrespective of the geographic region or local temperature, because deep-Mediterranean waters are relatively warm (14 °C on average) (Martin-Cuadrado et al. 2007). Also, all the genes that have been proposed to take part in the 3-hydroxypropionate/4-hydroxybutyrate cycle for autotrophic carbon fixation in *N. maritimus* (Walker et al. 2010) were present in the thaumarchaeal pangenome, reinforcing the idea that deep-sea planktonic Thaumarchaeota have the potential for chemolithoautotrophic growth. In contrast, despite a minimum of nine complete genomes were represented in the GII/III-Euryarchaeota data set, genes encoding proteorhodopsin homologs were not detected. This absence suggests that these GII-Euryarchaeota are genuine deep-sea dwellers that differ from their surface, proteorhodopsin-containing, counterparts (Frigaard et al. 2006; Iverson et al. 2012). They are most likely heterotrophic given the abundance of genes involved in amino acid, carbohydrate, and lipid transport and metabolism (see below). This is in agreement with deep-sea metatranscriptomic studies showing high levels of GII-Euryarchaeota amino acid transporter transcripts (Baker et al. 2013).

### Determining Categories of Core, Lineage-Specific Core, and Shell Genes in Archaeal Pangenomes

**Fig. 3.—:**
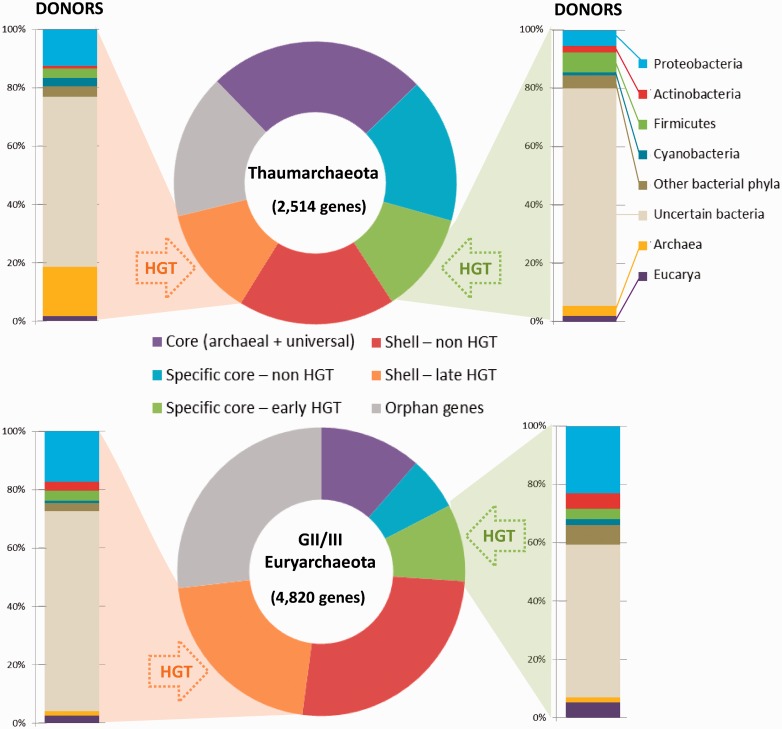
Distribution of genes in deep-Mediterranean Thaumarchaeota and GII/III-Euryarchaeota pangenomes as a function of their class of origin. The proportion of distant donors for early- and late-horizontally acquired genes is indicated.

Using fosmid sequences in metagenomic studies offers the advantage (shared with single-cell genomes when they are not too partial) of having access to sets of genes that are physically linked in a genome, therefore allowing the identification of accessory genes that are rare or present only in a subset of strains and that might be overlooked when reconstructing composite scaffolds from bulk short metagenomic sequences (Iverson et al. 2012). Starting from our fosmid sequences, we could thus define collections of OG clusters representing deep-Mediterranean Thaumarchaeota and GII/III-Euryarchaeota pangenomes. Subsequently, we classified them into core archaeal genes (universal genes and genes shared by all archaea), lineage-specific core genes (genes shared by, respectively, all—or all but one in the case of Thaumarchaeota, to accommodate single-lineage losses—archaeal genomes), and shell or accessory genes (only present in one or a reduced subset of genomes within each lineage) (see Materials and Methods). Excluding predicted genes with no homologs (orphans), a total of 2,098 and 3,527 OG clusters were identified, respectively, for the thaumarchaeal and GII/III-euryarchaeal pangenomes (table 1). Some of them were universal genes or genes shared by all archaea (629 and 552 for, respectively, thaumarchaeal and GII/III-euryarchaeal pangenomes). To define Thaumarchaeota-specific core genes, we considered OGs shared by our deep-Mediterranean fosmids and the genomes of their closest phylogenetic relatives from aquatic environments, namely, *N. maritimus* SCM1 (NC_010085), *C. symbiosum* A (NC_014820), and *N. limnia* SFB1 (Blainey et al. 2011) (fig. 1) to the exclusion of other archaea, which resulted in a total of 706 Thaumarchaeota-specific core genes. Likewise, for GII/III-Euryarchaeota, we used the composite genome built from surface seawater metagenome (CM001443.1) (Iverson et al. 2012), resulting in a remarkably similar number, 704, of GII/III-Euryarchaeota-specific core genes (table 1 and fig. 3). The remaining OGs present in only a subset of Thaumarchaeotal or GII/III-Euryarchaeota fosmids, having only one to three hits in the database or lacking homologs in archaea but not in other life domains were classified as shell genes. The total number of shell genes in GII/III-Euryarchaeota (2,271) was much larger than that of Thaumarchaeota (763).

### Phylogenetic Identification of Early and Late HGT Events

We incorporated to our deep-Mediterranean data set representative genomic sequences covering a comprehensive taxonomic sampling of archaea (including genomes, metagenomes, and environmental fosmids), bacteria, and eukaryotes and carried out phylogenetic analyses of all OGs. These were used to refine the definition and identification of archaeal core, lineage-specific core, and shell gene classes. Whenever the query OGs were robustly nested within bacteria, eukaryotes, or other distant archaeal phyla (see criteria to define HGT events in Materials and Methods), they were considered horizontally transferred genes (HT-genes).

**Fig. 4.—:**
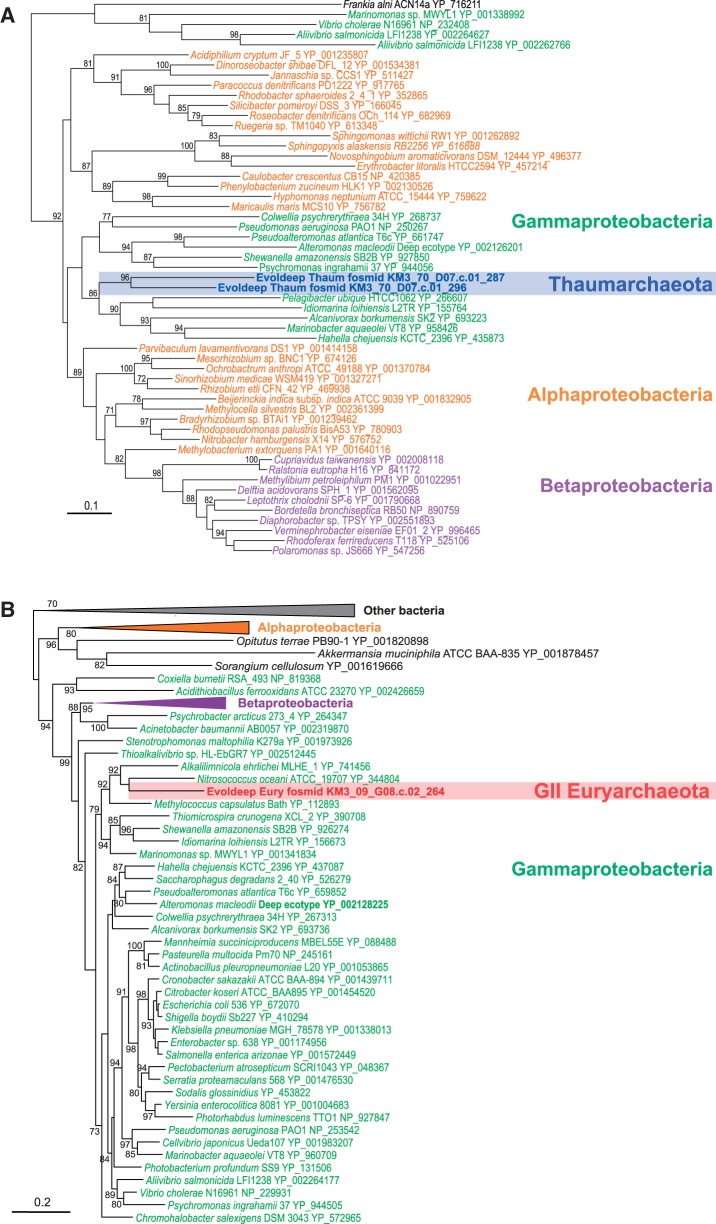
Maximum-likelihood phylogenetic trees of shell genes showing examples of late HGT from bacteria to deep-Mediterranean Thaumarchaeota (*A*) and GII/III-Euryarchaeota (*B*). (*A*) 6-Phosphogluconate dehydrogenase, 273 conserved amino acid positions. (*B*) Phosphoribosylamine-glycine ligase, 336 conserved amino acid positions.

**Fig. 5.—:**
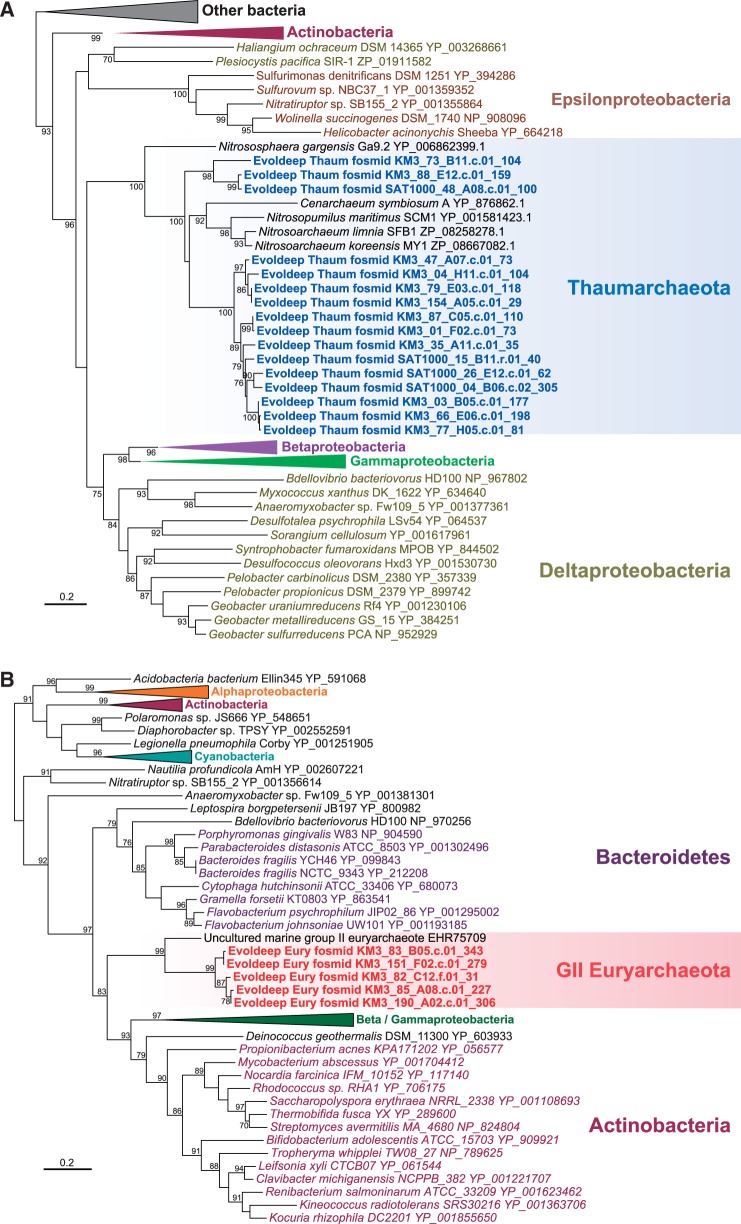
Maximum-likelihood phylogenetic trees of shell genes showing examples of early HGT from bacteria to deep-Mediterranean Thaumarchaeota (*A*) and GII/III-Euryarchaeota (*B*). (*A*) Methionine adenosyltransferase, 341 conserved amino acid positions. (*B*) Exodeoxyribonuclease III, 152 conserved amino acid positions.

We identified a high HGT level in the two archaeal-lineage pangenomes, amounting to 23.9% in Thaumarchaeota and 29.7% in GII/III-Euryarchaeota (table 1 and fig. 3). These HT-genes were found in lineage-specific core and shell gene classes. HT-genes in the shell fraction nested within distant donor lineages in phylogenetic trees but were absent from complete thaumarchaeal genomes or the composite marine surface GII-euryarchaeote (see examples in fig. 4). This implies that they were acquired by HGT from distant donors recently, after the diversification of Thaumarchaeota and GII/III-Euryarchaeota, respectively. Surprisingly, HGT events that occurred at the base of these two archaeal lineages were also abundant (fig. 3; see examples in fig. 5). Clear HGT affecting these archaeal genes could be inferred even if some cases of HGT among bacteria could sometimes be observed; the latter appears inevitable given the large phylogenetic scales considered. In Thaumarchaeota, they were as abundant (11.5%) as late HT-genes (12.6%). In GII/III-Euryarchaeota, they accounted for 8.6% (compared with 21.1% late HT-genes), although this proportion corresponds to a high number of genes (416) that might increase when representative true complete genomes become available for this lineage. Because our reconstructed thaumarchaeal pangenome included basal fosmids branching earlier than soil Thaumarchaeota, we would expect finding shared HT-genes in soil members. To test it, we looked for homologs of the HT-genes identified in our thaumarchaeal pangenome in *N. gargensis* (Spang et al. 2012) and reconstructed the corresponding phylogenetic trees. *Nitrososphaera gargensis* shared 196 HGTs out of the 290 genes that had been identified as early HT-genes in the Thaumarchaeotal pangenome (table 1).

Which were the distant donors of HT-genes? The majority were bacteria: 94% and 93% of early HT-genes and 81% and 96% of late HT-genes for deep-Mediterranean Thaumarchaeota and GII/III-Euryarchaeota, respectively (fig. 3). A very minor fraction came from eukaryotic donors or from other archaeal phyla (Euryarchaeota for the Thaumarchaeota or Crenarchaeota/Thaumarchaeota for the Euryarchaeota). Only recent HT-genes in Thaumarchaeota had a significant fraction of euryarchaeal donors (17%). Among the bacterial donors, between one-fourth and one-third of the HGT events could be ascribed to specific bacterial phyla, the remaining cases could not be confidently assigned to particular phyla. Proteobacteria, Actinobacteria, Firmicutes, and Cyanobacteria were the most frequently identified donors (fig. 3).

We checked that the high level of HGT in thaumarchaeal and GII/III-euryarchaeal fosmids from bacterial donors was not due to the inclusion in our analysis of chimeric archaeal/bacterial fosmids artificially produced during the fosmid library construction. First, our fosmids were carefully verified and lacked frameshifts that might be indicative of chimerism. Second, when we mapped the genes on fosmid-cloned genome fragments as a function of their origin class (archaeal core, lineage-specific core, early HT-genes, late HT-genes, and others), both early and late HT-genes were scattered among typical archaeal genes from the other classes. As a proxy to quantify this, we computed the mean synteny block length per gene class. In general, synteny blocks (here broadly defined as arrays of contiguous genes of same origin class) were small for all gene classes but those including early and late HGT events had the shortest average lengths (supplementary fig. S6, Supplementary Material online), indicating that HT-genes were transferred mostly as single genes and/or that HT-genes interspersed after transfer into host genomes. We also analyzed the class of origin of their flanking genes. Interestingly, the distribution patterns observed were very similar for the same gene classes defined independently of the archaeal phylum considered. Thus, early Thaumarchaeota HT-genes displayed a flanking pattern more similar to the corresponding class in GII/III-Euryarchaeota than to any other gene class in Thaumarchaeota, and so on (supplementary fig. S6, Supplementary Material online). This observation may be suggestive of similar histories for each gene class and/or similar evolutionary processes involved. We also looked for the presence of potential insertion elements, transposons, or viral sequences flanking HT-genes, but we failed to detect a clear association of such elements with HT-genes.

**Fig. 6.—:**
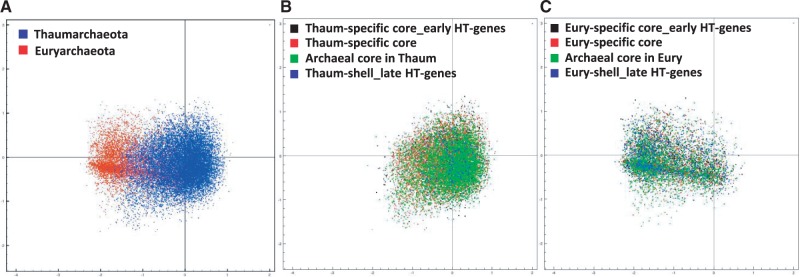
PCA of codon usage in deep-Mediterranean Thaumarchaeota and GII/III-Euryarchaeota pangenomes. (*A*) Genes colored as a function of their thaumarchaeal or euryarchaeal origin. (*B*) GII/III-euryarchaeal genes colored as a function of their class of origin. (*C*) Thaumarchaeal genes colored as a function of their class of origin.

Because differences in codon usage may be indicators of HGT (Garcia-Vallve et al. 1999), we looked for potential signatures of codon usage differences in recent HGT events when compared with other gene classes in deep-Mediterranean pangenomes. There were marked differences in codon usage between Thaumarchaeota and GII/III-Euryarchaeota pangenomes (fig. 6*A*) in agreement with manifest differences in GC content (table 1). However, differences in codon usage for recent HT-genes when compared with late HT-genes, lineage-specific core, or archaeal core genes in Thaumarchaeota (fig. 6*B*) or Euryarchaeota (fig. 6*C*) were not seen. Similar observations could be made from the CAI of the different gene classes considered. CAI measures the deviation of protein codon usage with respect to reference, highly expressed genes. All thaumarchaeal and GI/III-euryarchaeal gene classes had similar high CAI values when compared with their own reference data set (ribosomal proteins) (supplementary fig. S7, Supplementary Material online). This suggests that recent HGT events occurred sufficiently long ago for the corresponding genes to adapt to their host genomic environment.

### Functional Classes of Transferred Genes

**Fig. 7.—:**
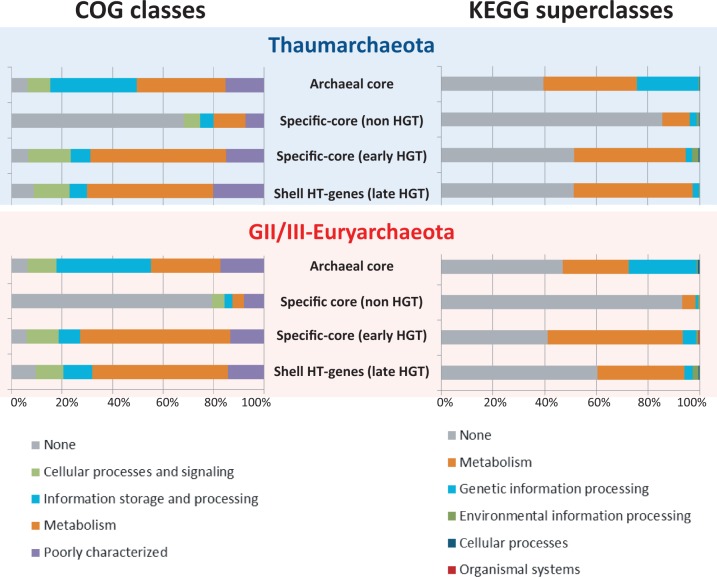
Distribution in COG classes and KEGG superclasses of deep-Mediterranean gene clusters according to their phylogenetic classification into archaeal core, lineage-specific core (non-HT-genes), early HT-genes, and accessory genes. Thaum, Thaumarchaeota; Eury, Euryarchaeota; and HTG, horizontally transferred genes.

We looked for potential functional differences between late and early HT-genes to Thaumarchaeota and GII/III-Euryarchaeota pangenomes, and between these and the corresponding archaeal core and lineage-specific core genes. Shell non-HGT genes were not included in this analysis, because they correspond to predicted genes with one to a few homologs only in fosmids and lacking clear homologs in the database (hence, nonannotated). Overall differences were already seen at a very general level of functional classification in COG classes and KEGG superclasses between gene origin classes. However, there were remarkable similarities in the functional patterns observed for the different gene origin classes between Thaumarchaeota and GII/III-Euryarchaeota (fig. 7), suggesting similar underlying processes and/or mechanisms of adaptation by gene acquisition. These similarities were significant for all COG distributions and for all the KEGG distributions except that of late HT-transfers (supplementary table S1, Supplementary Material online). As expected, archaeal core (including universal) genes in the two lineages contained the most important fraction of genes involved in storage and processing of genetic information, together with an equivalent (or slightly smaller) fraction of genes involved in metabolism. Early and late HT-genes also displayed a remarkable similarity. Their COG classes were clearly dominated (ca. 60%) by metabolism-related genes, although also included a few informational and signaling-related genes. Whenever classifiable, their KEGG superclasses were also largely dominated by metabolism-related genes; their levels were very similar in Thaumarchaeota (ca. 50%), although a slight difference was observed between early HT-genes (ca. 55%) and late HT-genes (ca. 35%) in GII/III-Euryarchaeota. However, the most striking difference corresponded to the patterns displayed by Thaumarchaeota- and GII/III-Euryarchaeota-specific core genes, which were clearly dominated by genes that could not be attributed to existing COG classes (70–80%) or KEGG superclasses (ca. 90%), the remaining fraction being dominated by metabolism-related genes.

At finer scale, some differences were observed between early and late HT-genes and between Thaumarchaeota and GII/III-Euryarchaeota in COG categories (supplementary figs. S8 and S9, Supplementary Material online). However, the general functional categories most affected by HGT were similar in the two lineages: Nucleotide, coenzyme, carbohydrate, lipid and amino acid transport and metabolism, inorganic ion transport, energy production and conversion, and cell wall/membrane biogenesis. Late HT-genes contained more genes with general prediction only. GII/III-Euryarchaeota appeared more impacted by HGT and contained more HT-genes affecting transcription/signal transduction and posttranslational modifications than Thaumarchaeota.

The distribution of HT-genes that could be assigned to KEGG pathways also revealed that early and late HGT events distributed similarly in each of the lineages, with ancient HT-genes being more represented than recent HT-genes in the identified pathways. The only exceptions were the import of lysine and streptomycin biosynthesis in GII/III-Euryarchaeota (supplementary fig. S10, Supplementary Material online). Likewise, there were some similarities between the global distribution of HT-genes in Thaumarchaeota and GII/III-Euryarchaeota, which were high in functions such as benzoate degradation, phenylalanine metabolism, folate biosynthesis, fatty acid biosynthesis, ABC transporters, oxidative phosphorylation, or the metabolism of glyoxylate and dicarboxylate, cysteine, methionine and, to some extent, other amino acids (although with variations in percentages), and cofactors. However, there were also important differences. Thaumarchaeota seem to have imported more genes related to sugar metabolism (fructose, mannose, galactose, aminosugar, and nucleotide sugar metabolism), whereas GII/III-Euryarchaeota seem to have acquired more genes involved in amino acid and nucleotide metabolism (HT-genes related to streptomycin and lysine biosynthesis; the pentose phosphate pathway; and the metabolism of thiamine, pyruvate, or nitrogen, fatty acids, alanine, aspartate and glutamate, and pyrimidine).

## Discussion

HGT is an important force in evolution, contributing to innovation and adaptation to changing or new environments through the expansion of gene families and the import of radically different metabolic functions (Ochman et al. 2000; Gogarten et al. 2002; Treangen and Rocha 2011). Our work shows that this likely applies to two different lineages of planktonic mesophilic archaea whose members remain largely uncultured, deep-sea Thaumarchaeota, and GII/III-Euryarchaeota. Using metagenomic fosmid libraries from deep-Mediterranean plankton, we were able to build comprehensive pangenomes for these two diverse archaeal lineages and to show, by phylogenetic analyses of all OGs, that HGT is an extensive phenomenon, with 23.9% (Thaumarchaeota) and 29.7% (GII/III-Euryarchaeota) of genes having been acquired in this way from distant donors, essentially bacteria. This level of HGT is in agreement with previous estimates based on a few fosmid and fosmid-end sequences (Brochier-Armanet et al. 2011). Even if our estimates of HGT seem high, they are indeed conservative, because we could only determine with confidence HGT cases from sufficiently resolved phylogenetic trees. Given the extent of HGT in conserved genes, it seems reasonable to hypothesize that an unknown fraction of less-conserved genes and/or genes for which sampling was too poor, which were dismissed in our analysis, might have also been acquired by HGT from distant donors. This “long-distance” HGT phenomenon is ongoing and does not affect only shell genes (recent HT-genes) but also lineage-specific core genes. This implies that a significant fraction of genes were acquired by the ancestors of marine Thaumarchaeota and GII/III-Euryarchaeota, respectively, and that a significant fraction of these transfers was also vertically inherited by the soil Thaumarchaeota branching off the marine clade (*Nitrososphaera* sharing a large fraction of those HT-genes). Although the fraction of early HT-genes was not apparently as high in GII/III-Euryarchaeota (ca. 9%), it corresponded to a prominent number of genes (416 genes; table 1) and might simply reflect the higher number of genes defined as shell. Indeed, the definition of the GII/III-Euryarchaeota pangenome was based in shared genes with a genome scaffold reconstructed from bulk short metagenome sequences (Iverson et al. 2012), which might favor the elimination of accessory genes not shared by all strains. In fact, some genes identified as late HT-genes could change to the early HT-genes as more GII/III-euryarchaeal genomes become available.

The relative high level of HGT found in Thaumarchaeota and GII/III-Euryarchaeota genomes supports the idea that shared HGT events can be used as support for the monophyly of prokaryotic lineages (Abby et al. 2012) and suggests that HGT from bacteria has been an important determinant in the evolution of those two archaeal lineages. In recent comparisons of COGs in archaeal genomes, Wolf et al. (2012) inferred a gain of 494 genes at the base of the Thaumarchaeota while asserting that most gene gain should be derived from HGT. Our direct observations confirm that prediction to a large extent, since we observed 290 cases of early HGTs to the ancestor of marine and soil Thaumarchaeota. Although slightly inferior, gene gain can also occur from gene duplication and de novo formation. In addition, some of the late HT-genes that we observe may be early HT-genes followed by losses in some specific lineages. Such losses would be indeed consistent with the expected streamlined nature of deep-sea archaea living in oligotrophic conditions. They might also explain the ongoing nature of HGT in these archaea, in eventual agreement with the hypothesis that HGT is a need in lineages under genome size constraint (Isambert and Stein 2009).

HGT in deep-sea Thaumarchaeota and GII/III-Euryarchaeota is not only extensive and ongoing but also directional, with most HT-genes having been imported from bacteria. This confirms a trend already observed in cases of interdomain HGT, which mostly occur from bacteria to archaea and not the opposite (Kanhere and Vingron 2009; Nelson-Sathi et al. 2012). The high level of bacteria-to-archaea HGT might lead to several, nonmutually exclusive, hypothetical explanations. First, because bacteria dominate in terms of both diversity and relative abundance in most environments, including oceans, preferential bacteria-to-archaea HGT might be simply a statistical outcome. Second, archaea might have a higher capacity to incorporate foreign genes, for instance, through facilitated gene import and genome incorporation via known and/or yet-to-be discovered mechanisms and keep them if these are of adaptive value. Third, archaea might experience a lower cost of HGT in terms of fitness, implying an easier fixation of HT-genes. Lower fitness costs would depend on how the genomic environment accommodates foreign DNA (Baltrus 2013) and on the “friendliness” of HT-gene products (Gophna and Ofran 2011). Finally, an additional explanation might be related to the adaptive benefits that the newly acquired genes provide. In this sense, genes related to metabolism and providing new functions should be enriched in HT-genes. Exploring the potential contribution of these different factors should help to understand the underlying mechanisms of genome evolution in archaea.

From a functional point of view, our pangenome results reinforce the idea that deep-sea Thaumarchaeota are ammonia oxidizers able to metabolize urea with a potential for chemolithoautotrophic growth. Deep-sea GII/III-Euryarchaeota seems to be heterotrophic organisms lacking the photoheterotrophic ability of their proteorhodopsin-containing surface relatives. Many of the genes involved in the metabolic function of these lineages may be genuinely archaeal. Indeed, one striking observation corresponds to the high level of lineage-specific core genes of unknown function, which contrasts to HT-genes and highlights how little is known about the function of lineage-specific core genes in these archaea (fig. 7). Nevertheless, metabolism-related genes are the most abundantly acquired by HGT in Thaumarchaeota and GII/III-Euryarchaeota (fig. 7). In particular, the large proportion of HT-genes related to membrane biogenesis in our thaumarchaeal and GII/III-euryarchaeal pangenomes (supplementary figs. S8–S10, Supplementary Material online) suggests that at least an important fraction of functions related to membrane activity and recognition, which are of uttermost importance in cold, oligotrophic oceans, have been imported form bacteria.

## Supplementary Material

Supplementary files S1 and S2, table S1, and figures S1–S10 are available at *Genome Biology and Evolution* online (http://gbe.oxfordjournals.org/).

Supplementary Data
