# Supplementary material for: Pangenome Evidence for Extensive Interdomain Horizontal Transfer Affecting Lineage Core and Shell Genes in Uncultured Planktonic Thaumarchaeota and Euryarchaeota
Source: Genome Biol Evol. 2014 Jun 12;6(7):1549–63. doi: 10.1093/gbe/evu127 (PMC4122925; doi:10.1093/gbe/evu127)
Supplement: Supplementary Data [file supp_6_7_1549__index.html]

Pangenome evidence for extensive inter-domain horizontal transfer affecting lineage-core and shell genes in uncultured planktonic Thaumarchaeota and Euryarchaeota — Pangenome Evidence for Extensive Interdomain Horizontal Transfer Affecting Lineage Core and Shell Genes in Uncultured Planktonic Thaumarchaeota and Euryarchaeota — Supplementary Data 

# Pangenome Evidence for Extensive Interdomain Horizontal Transfer Affecting Lineage Core and Shell Genes in Uncultured Planktonic Thaumarchaeota and Euryarchaeota

## Supplementary Data

files

**Files in this Data Supplement:**

- Supplementary Data - zip file
