# Supplementary material for: Pangenome Evidence for Extensive Interdomain Horizontal Transfer Affecting Lineage Core and Shell Genes in Uncultured Planktonic Thaumarchaeota and Euryarchaeota
Source: Genome Biol Evol. 2014 Jun 12;6(7):1549–63. doi: 10.1093/gbe/evu127 (PMC4122925; doi:10.1093/gbe/evu127)
Supplement: Supplementary Data [file supp_evu127_suppl_data.zip › Deschamps-etal_Supplementary information.pdf]

Table S1. Chi-square tests for distributions of gene classes in Thaumarchaeota as compared to GII/III-Euryarchaeota. HT-genes, horizontally transferred genes. *, only case for which the two distribution patterns were not significantly similar.

	**COG**	**KEGG**
Thaumarchaeota vs. GII/III Euryarchaeota archaeal core	0.301596376	0.123338621
Thaumarchaeota vs. GII/III Euryarchaeota-specific core non HT-genes	0.097462121	0.196948972
Thaumarchaeota vs. GII/III Euryarchaeota-specific HT-genes (early transfers)	0.544409189	0.050684926
Thaumarchaeota vs. GII/III Euryarchaeota shell HT-genes (late transfers)	0.324156576	4.03236E-07*

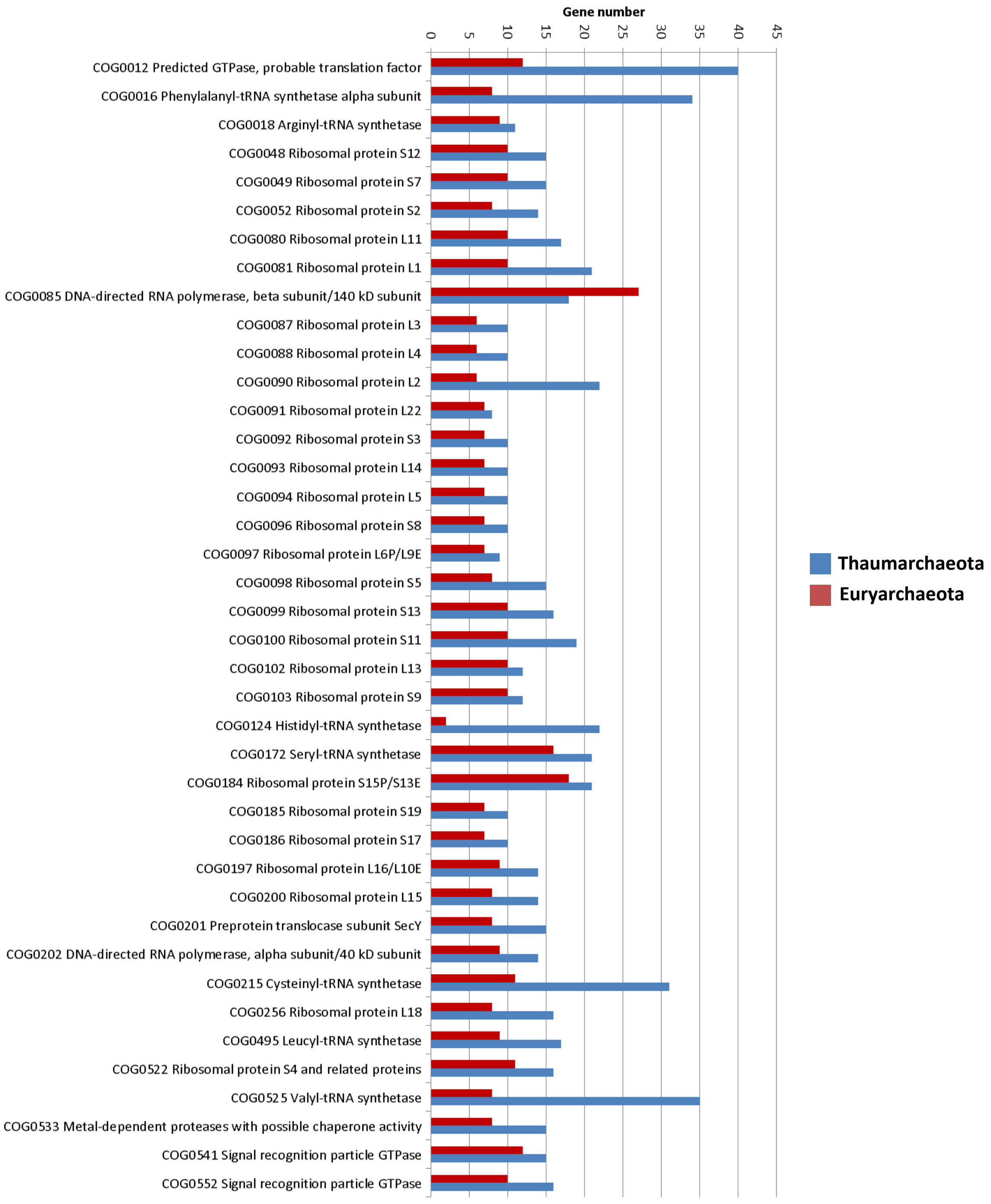

Fig. S1. Number of 40 genes usually found in single copy in prokaryotic genomes (Creevey et al, 2011) present in genomes of deep-Mediterranean Thaumarchaeota an GII-Euryarchaeota.

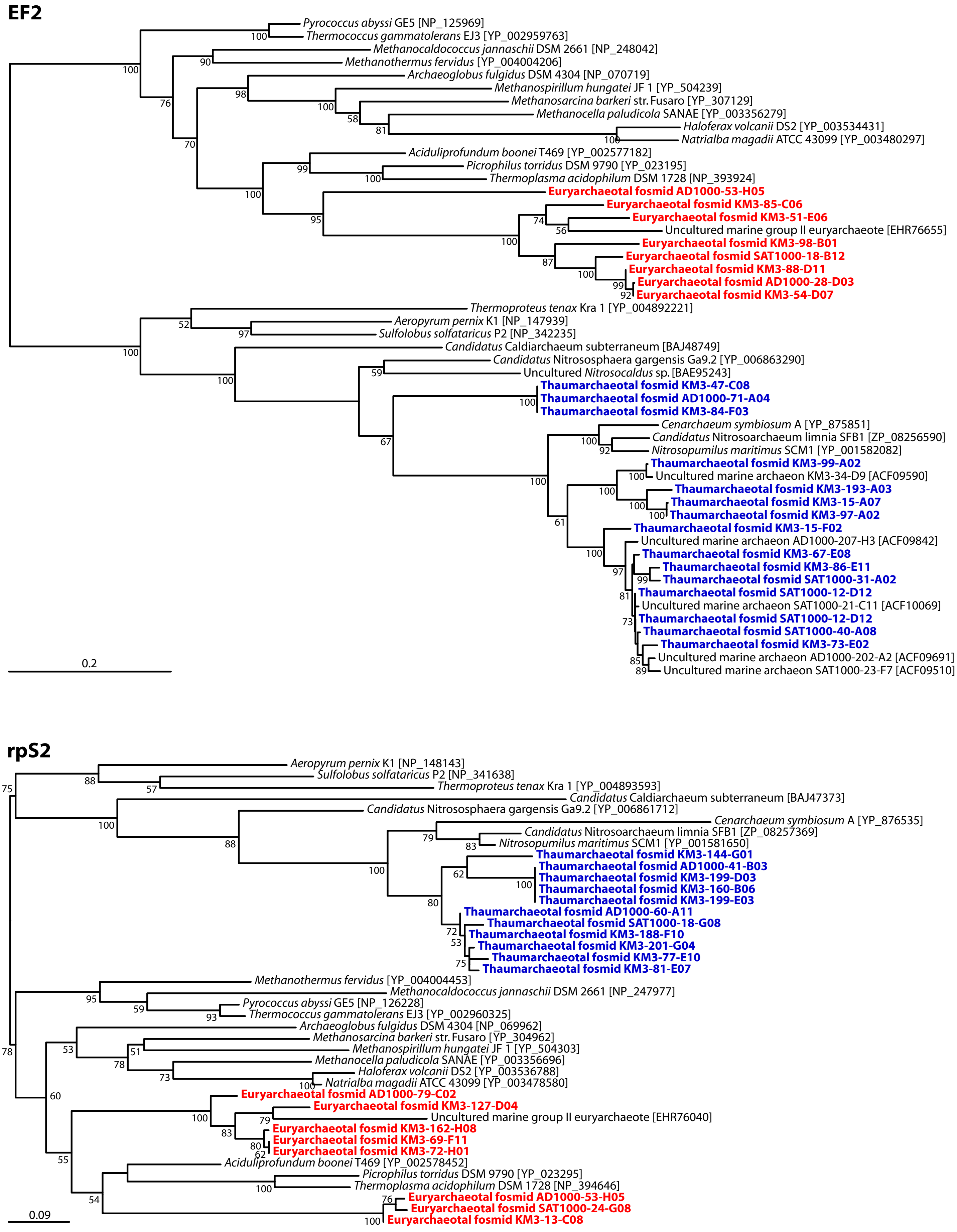

Fig. S2. Phylogenetic trees of elongation factor 2 (EF2) and ribosomal protein S2 genes present in deep-Mediterranean archaeal fosmids.

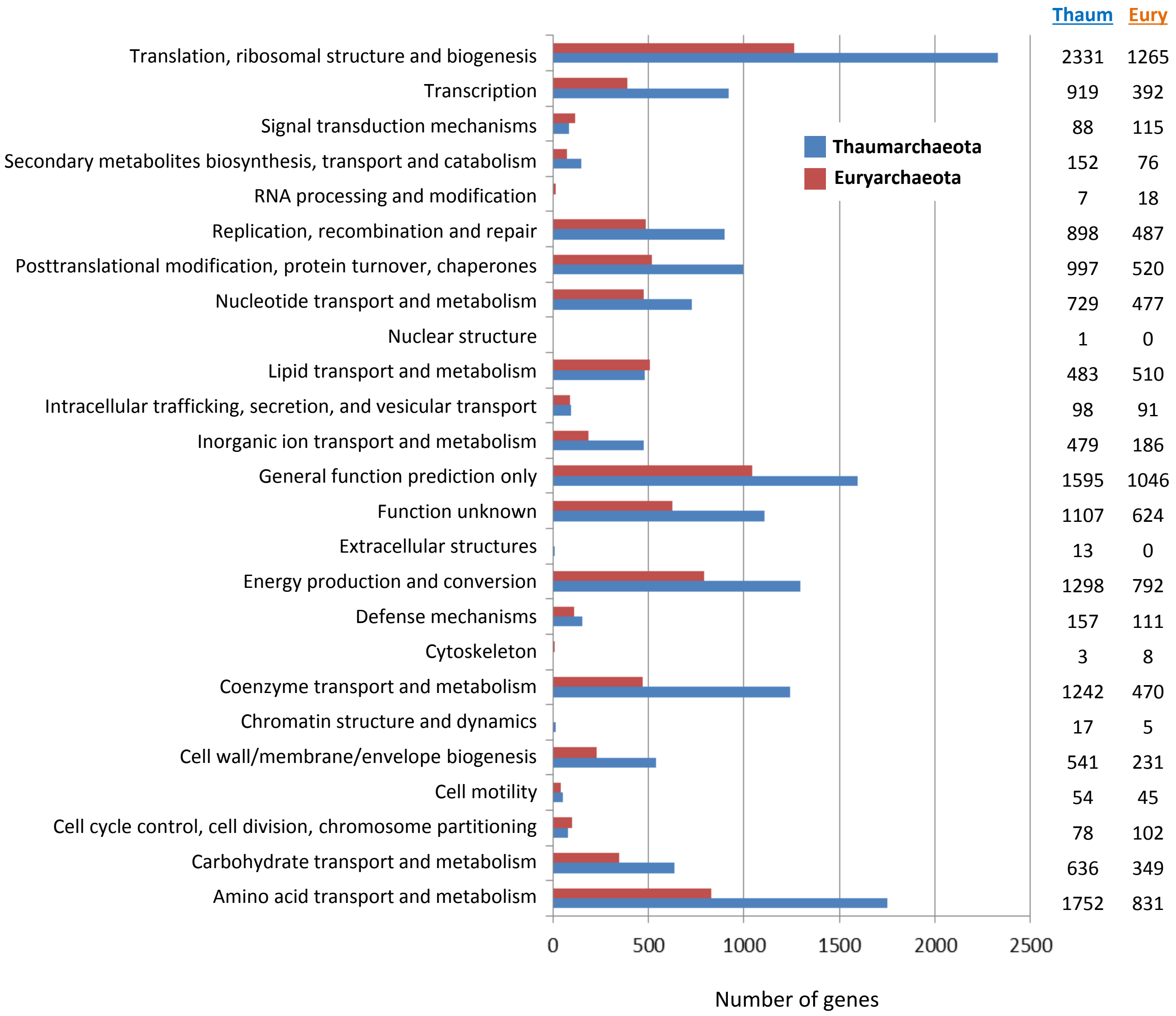

Fig. S3. Comparative distribution of genes in COG categories for the Thaumarchaeota (blue) and the Group II/III Euryarchaeota (red) in deep-Mediterranean archaeal fosmid metagenome sequence. Total gene counts are given on the right.

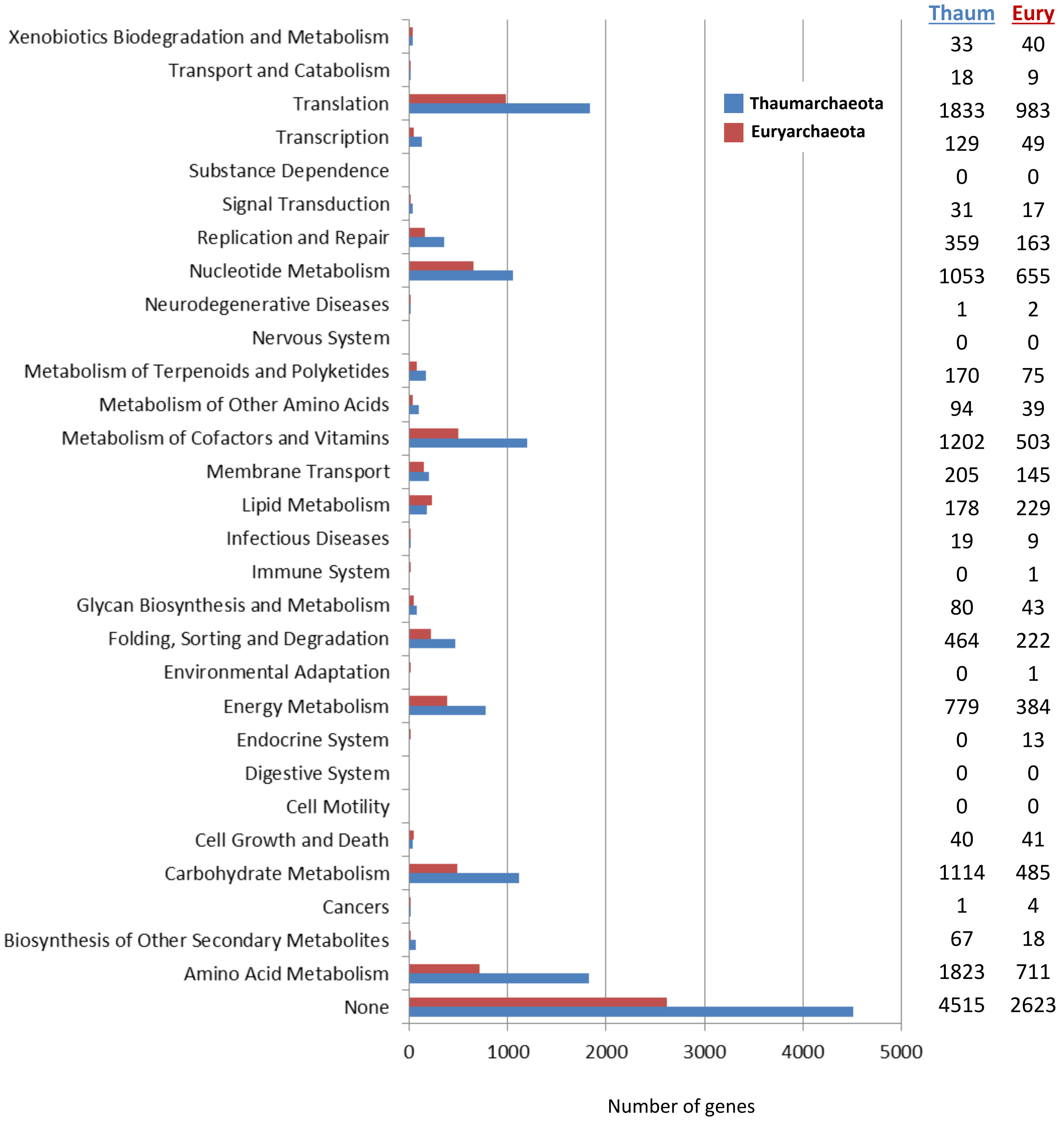

Fig. S4. Comparative distribution of genes in KEGG classes for the Thaumarchaeota (blue) and the Group II/III Euryarchaeota (red) in deep-Mediterranean archaeal fosmid metagenome sequence. Total gene numbers are given on the right.

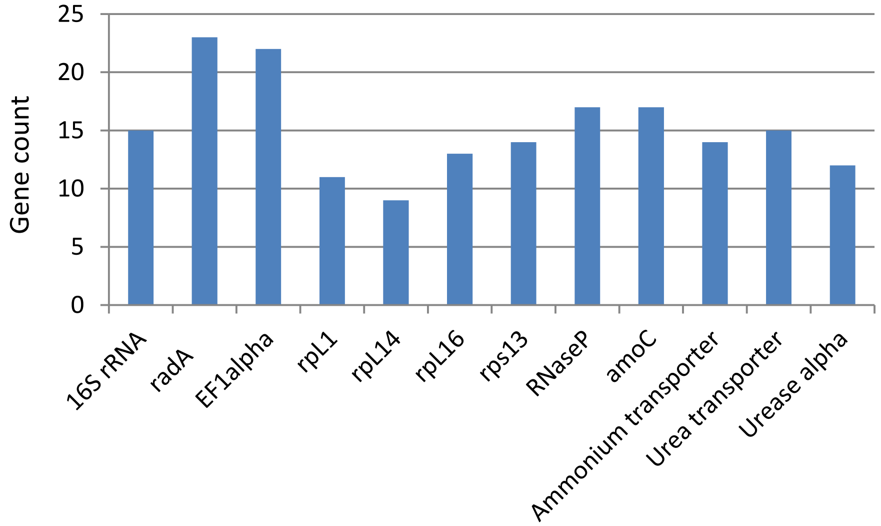

Fig. S5. Number of genes related to ammonium and urea metabolism in deep-Mediterranean Thaumarchaeotal genomes as compared to the number of typical single copy genes.

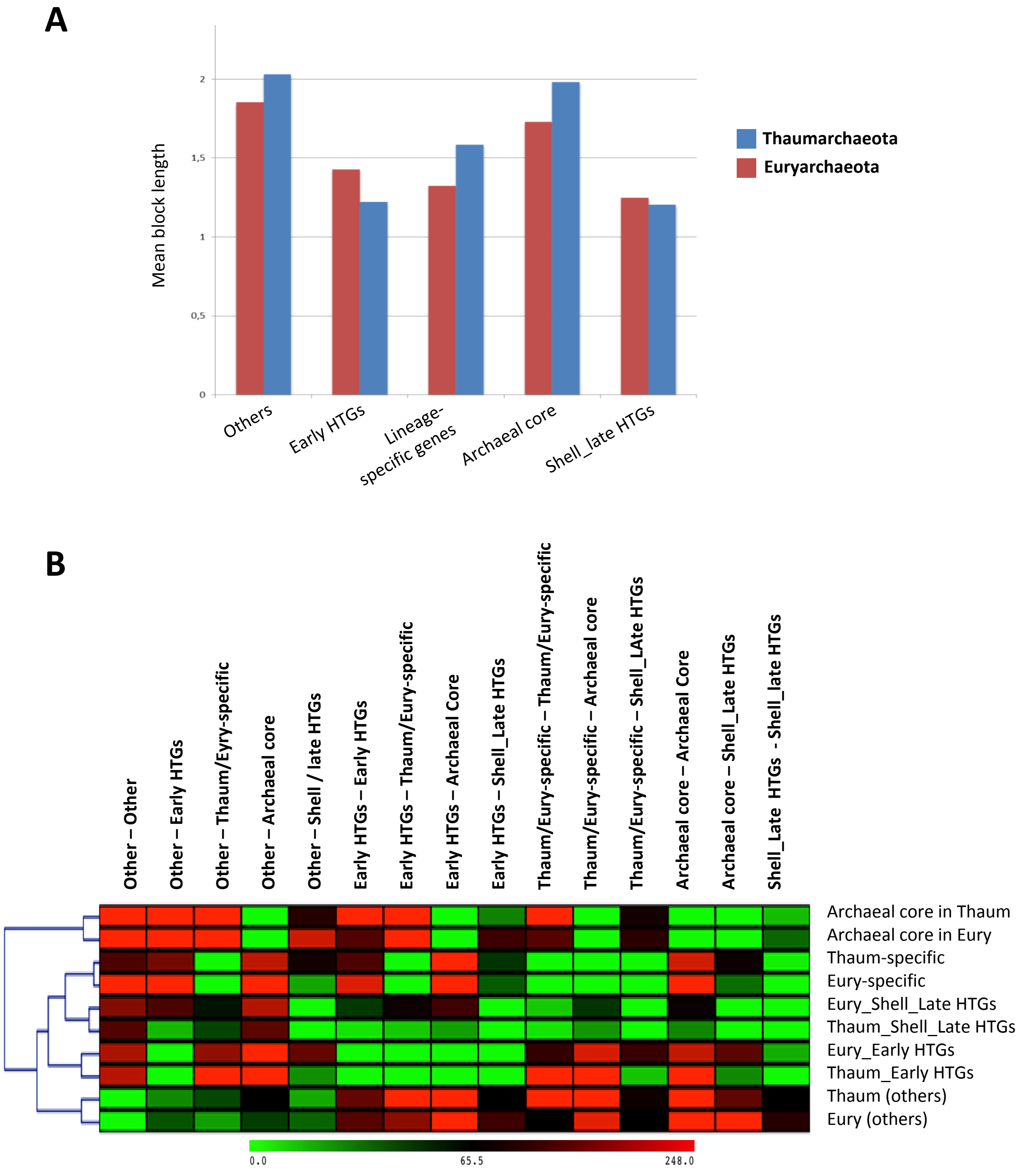

Fig. S6. Synteny proxies per classes of gene origin in Thaumarchaeota (Thaum) and GII/III-Euryarchaeota (Eury) genomes. **A**, mean synteny block length per block origin. **B**, cluster of synteny blocks as a function of block bounds. A block is defined as an array of contiguous genes from same origin (class). 'Others' includes orphans plus non-HGT shell genes.

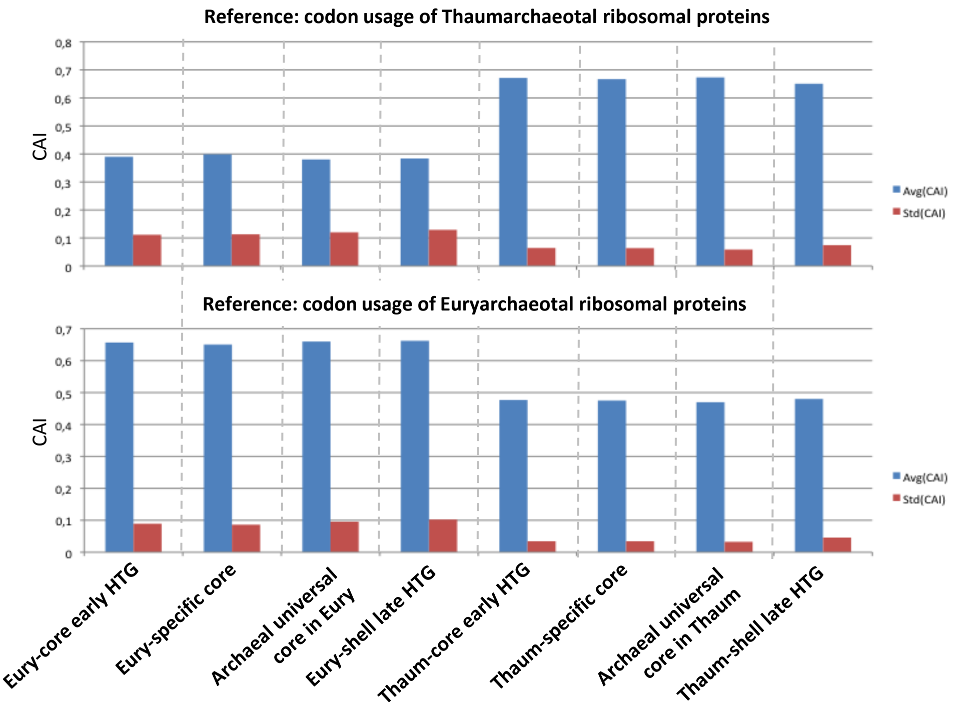

Fig. S7. Codon adaptation index (CAI) of different gene classes in deep-sea Thaumarchaeota and GII/III-Euryarchaeota as referred to the codon usage of thaumarchaeotal (Thaum) or GII/III-euryarchaeotal (Eury) ribosomal genes. Avg, average value; Std, standard deviation. HTG, horizontally transferred genes.

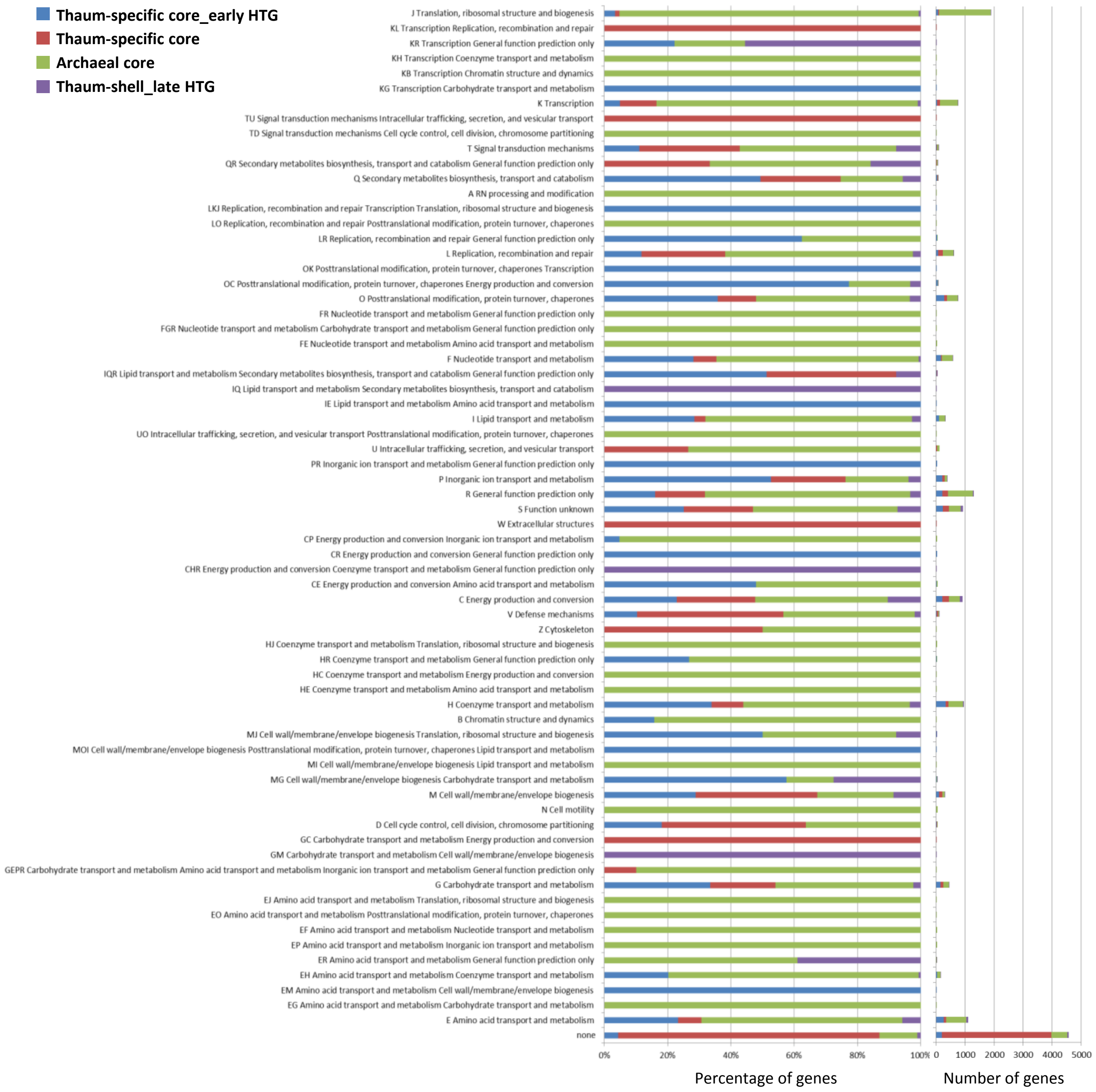

Fig. S8. Distribution of gene clusters of deep Mediterranean Thaumarchaeota in archaeal core, Thaumarchaeota-specific core, Thaumarchaeota early HTG and accessory (shell) HTG distributed in COG categories.

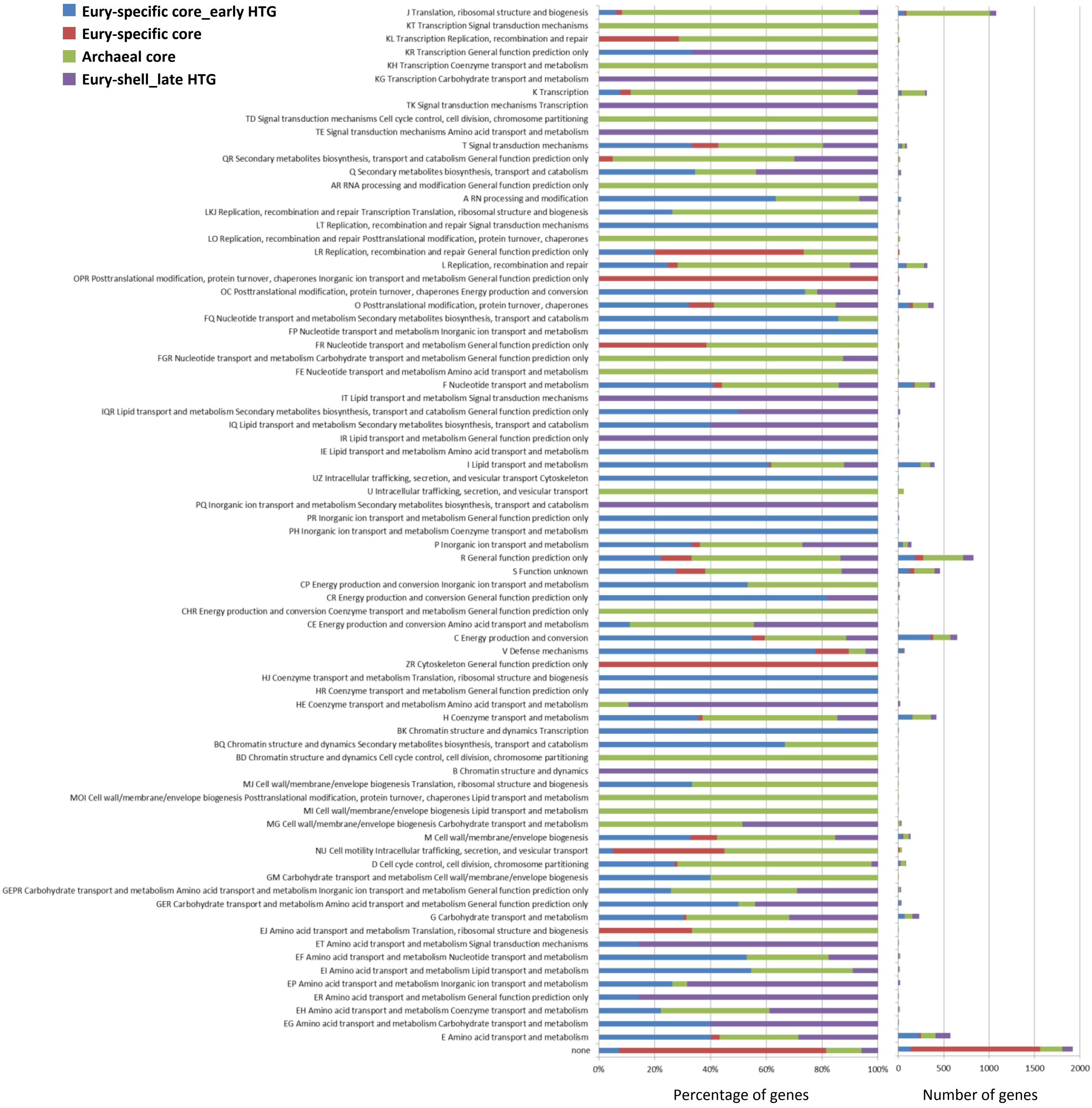

Fig. S9. Distribution of gene clusters of deep Mediterranean GII/III Euryarchaeota in archaeal core, Euryarchaeota-specific core, Euryarchaeota early HTG and accessory (shell) HTG distributed in COG categories.

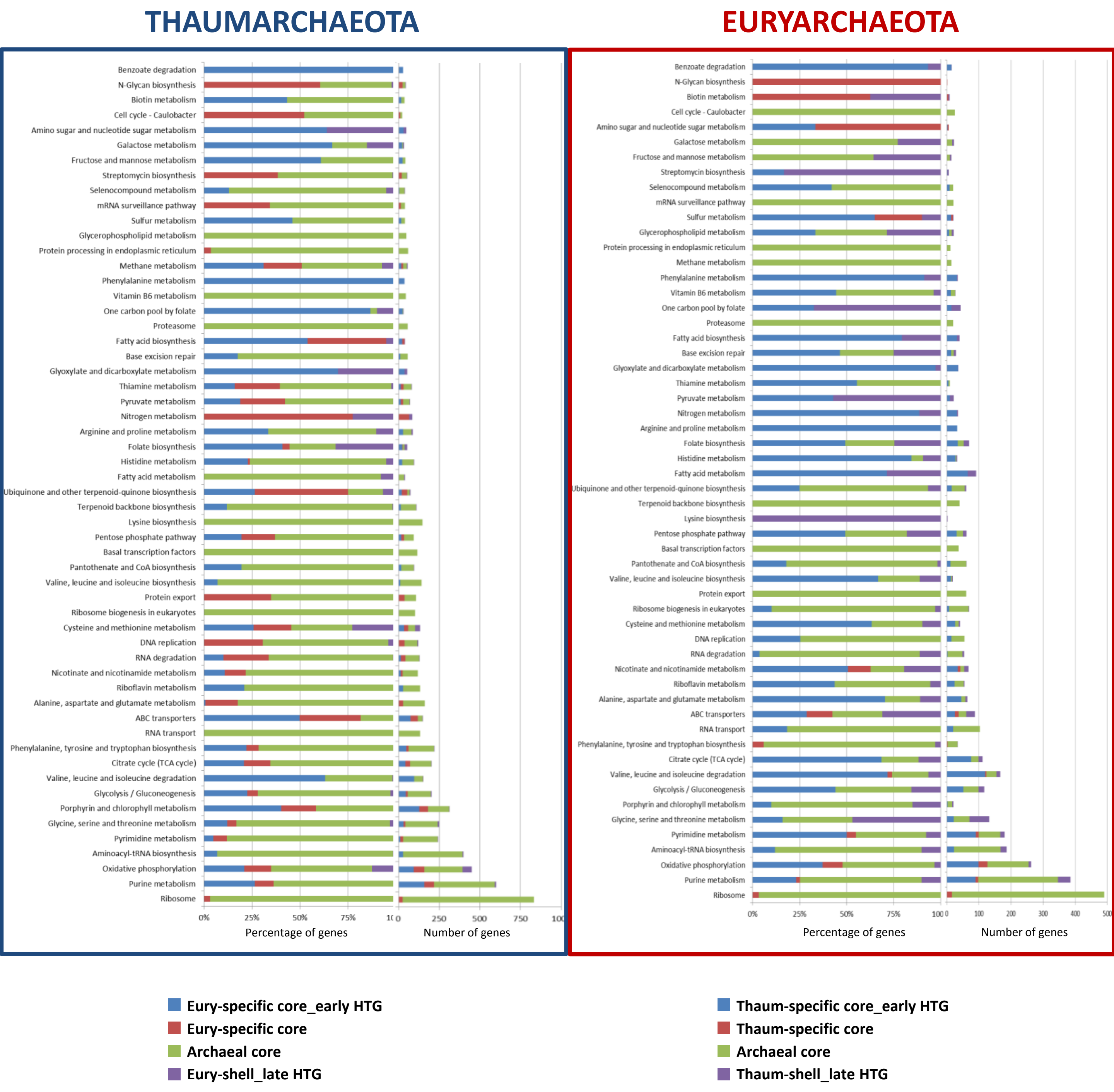

Fig. S10. Distribution of gene clusters (archaeal core, lineage-specific core, early HTG and accessory genes) distributed in KEGG pathways. Only pathways represented by more than 40 gene copies are included.
